# Squamous cell carcinoma related with dental implants. A clinical cases report

**DOI:** 10.4317/jced.55964

**Published:** 2020-01-01

**Authors:** Francisco Granados, Leonor Santos-Ruiz, Marian Contreras, Jose Mellado, Gregorio Martin, Lucas Bermudo, Francisco Ruiz, Yolanda Aguilar, Ignacio Yáñez

**Affiliations:** 1 University General Hospital of Málaga. Plaza de Hospital Civil, Málaga

## Abstract

One third of all cases of head and neck carcinoma (CA) concern the oral mucosa. The use of dental implants (DI) for dental rehabilitation is widely extended. However, a few studies have reported some cases with neoplasic alterations, among the tissue surrounding implants. Our aim was to analyze possible alterations at the bone-implant interface in patients with oral squamous cell carcinoma (SCC), providing new evidence that could relate or discard a possible link between these factors. We used, for the first time, different techniques, including electron microscopy and histology, to analyze the implant ´s surface and the surrounding tissue from four clinical cases with neoplasic alterations surrounding DI. Histologically, ample inflammatory tissue was found in direct contact with the implant surface. Surface analysis of this tissue, revealed titanium percentages. According to our study, no oncological relation with deterioration of the implant surface was found, although DI were constantly related with peri-implantitis, a chronic trauma of the oral mucosa that could involve a neoplastic factor.

** Key words:**Dental implants, carcinoma, peri-implantitis.

## Introduction

Head and neck cancer constitutes 3-5% of all malignancies, with 33% appearing over the oral mucosa, representing the most frequent histological type accounting for 90% of all cancers in the oral cavity (1). CA of the oral mucosa mainly affects men from the age of 50 years and is related to chronic tobacco and alcohol consumption, risk factors that are time and dose dependent. Nevertheless, recent years have seen an increase in this type of cancer among younger patients without these two classical risk factors. Thus, new risk factors are being sought to discover links with the carcinogenesis of SCC of the oral mucosa, such as certain agents that are irritant to the oral mucosa, like poor oral hygiene, poor dentition, ill-fitting dentures and missing teeth with the corresponding dental implants(DI) as mentioned by singhvi *et al*. and Mendes *et al.* (2,3).

Since the introduction of DI around 50 years ago (Branemark, 1965), DI have been used as a rehabilitation technique in patients treated for CA of the oral mucosa, who frequently suffer from surgical sequelae (2,4). Success rates of DI far outnumber failures and severe complications (3), the most frequent of which in implant surgery are related to the inflammatory process or peri-implantitis, a long-standing irritation factor (2). Though oral carcinoma in relation to DI has been increasing ( Table 1), with 49 clinical cases until 2016(69% primary tumors and 9.4% metastatic), with the majority concerning SCC (5-7). No carcinogenic role of DI is established; but different theories have been postulated, including corrosion of DI´s surface, release of metal ions, migration of malignant cells around gingiva and chronic inflammatory processes as peri-implantitis (6).

**Table 1:**
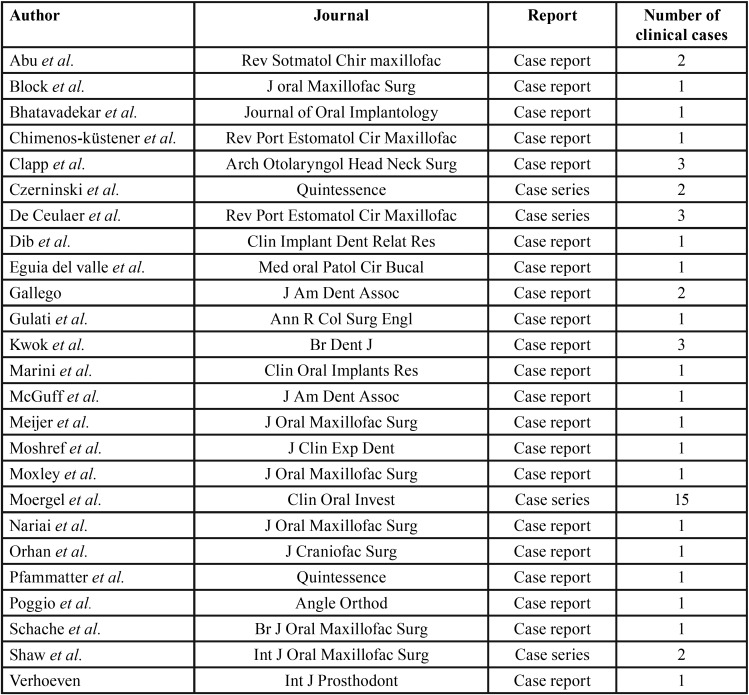
Summary of oral cancer cases related to DI published.

Case Report

Case 1: An 83-year-old man underwent bilateral cervical functional dissection and right partial mandibulectomy at our facility due to squamous cell carcinoma of the gums (T4N0Mx), receiving adjuvant radiotherapy. During routine clinical surveillance, a new ulcerous lesion was detected, surrounding the DI placed for rehabilitation eight years earlier. An implantoplasty (the use of a burr to smoothen rough implant surfaces which are exposed to the oral cavity, for reducing the adherence of bacterial plaque) was performed to eliminate contact between the implant and this lesion. Nevertheless, no improvement was noted after 6 months. A CT scan showed lytic lesions on the left mandibular body and lytic lesions on the cortical lingual side, surrounding the DI. A biopsy revealed well-differentiated invasive SCC and the patient underwent a segmental mandibulectomy, with a second radiotherapy regimen.

Case 2: A 60-year-old man reported a two-month history of a verrucous lesion on the gums, associated with pain and bleeding to the touch; over the alveolar rim of the third quadrant, located distal to a DI at position 37. Left cervical lymphadenopathy was palpable, level IIA. A CT scan and orthopantomography showed lytic lesion. Biopsy showed well-differentiated SCC, clinical stage T4N1Mx. A bilateral functional cervical dissection with segmental mandibulectomy was performed. Adjuvant radiotherapy and chemotherapy were administered.

Case 3: A 54-year-old woman with a mandibular dental prosthesis supported on DI with a two-month history of a gum lesion over the right mandible, and no cervical lymphadenopathies. CT scan showed bone resorption at mandibular symphysis adjacent to the gum lesion. After a biopsy confirmed the presence of SCC, we performed a bilateral cervical dissection with segmental mandibulectomy and micro-vascularized fibula free flap. Adjuvant radiotherapy was then given. 

Case 4: A 64-year-old man with diabetes reported an excrescent lesion over the mandibular vestibular gingiva, in contact with a dental prosthesis over two DI placed two years previously. A biopsy revealed poorly-differentiated SCC. A CT scan and orthopantomography showed a lytic bone area related to a soft tissue mass, corresponding to oncological stage pT4N0Mx, close to the DI. A bilateral cervical dissection and marginal mandibulectomy were performed, followed by adjuvant radiotherapy. Six months later the patient died due to metastatic spread of a lung’s adenocarcinoma, developed after oral CA.

Fragments of the resected mandibles, including the implants, were analyzed by Scanning Electron microscopy (SEM) with a JEOL JSM-6490LV SEM. A new, clean and sterilized DI, never implanted, was used as a control.

For histological evaluation the samples of the resected mandibles not containing the metal DI were decalcified, and embedded in paraffin. Sections were stained with Hematoxylin-Eosin. Samples containing the DI were not decalcified and stained with von Kossa’s Stain, to highlight bone, or with Toluidine Blue, to reveal soft tissues. All sections were observed and scanned with an Olympus VS120 microscope.

**Figure 1:**
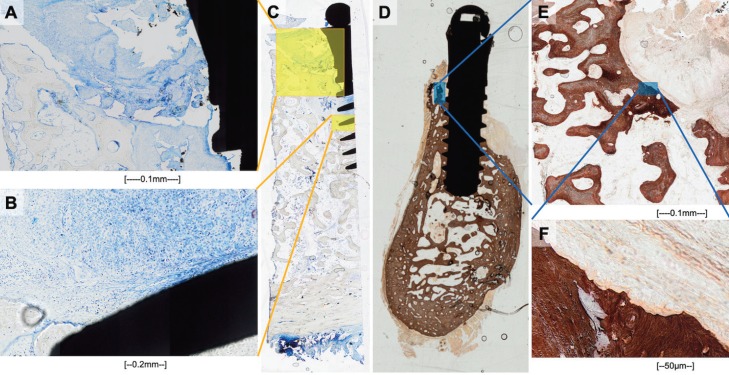
Toluidine’s blue stain, with a DI embedded into a cross section of mandible from pathologic sample. A, B: amplification image from tissue surrounding DI, with bone matrix and numerous leucocytes from inflammatory reaction. D, E and F: Von Kossa stain revealing peri-implant bone and inflammatory tissue, from the same sample as figures A and B. Revealing inflammatory response with bone resorption.

**Figure 2:**
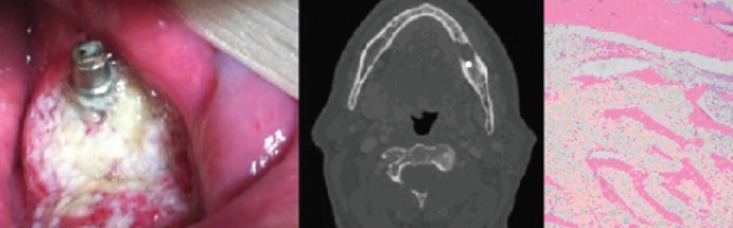
Clinical, radiological and histological images (hematoxylin-eosin dye) from case 2, depicting oncological lesion on the mandible around DI on third quadrant.

Histological analysis confirmed the episodes of peri-implantitis. As shown in Figures 1A and 1B (Toluidine’s Blue), an inflammatory tissue was present around the implant. This inflammation was associated with bone resorption, as observed by von Kossa staining (Figures 1C and 1D). SCC was found next to the inflammatory tissue on clinical examination (Fig. 2).

**Figure 3:**
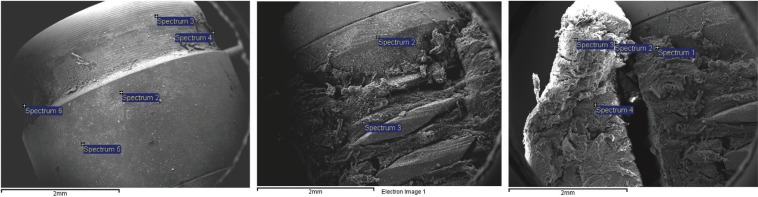
SEM images of dental implant surface (commercial implant on the left) and DI with organic osseous components from analyzed cases (center and right images).

Results show the control implant (Fig. 3 left) with Titanium (Ti) values close to 100%, as expected with a small percentage of Carbone (C) close to 4%. Calcium (Ca) and Oxygen (O) percentages where null, as this DI was never in contact with human tissue. All other DI exposed to human tissue, revealed Ti percentage on the tissues around the DI never higher than 1,5% (Fig. 3 right) Surface analysis of the DI had not less than 93% of Ti, and virtually no C. An exception was found in case 1, where Ti values over DI surface were between 22,07% and 54,83% (Fig. 3 center), with a greater percentage of C 30,98%This alteration in the superficial composition of the DI might have been due to the mechanical alteration reported in this case (see case 1). Ca and O where only found in the host’s tissue around DI, whereas C and Ti percentages where higher on DI surface.

## Discussion

Numerous carcinogenicity theories have been proposed, including corrosion of DI and release of Ti particles, that might be associated with cancer formation (6). However, according to Bhatavadekar *et al.*, no association had been demonstrated. We have performed, for the first time, SEM surface analysis to reveal possible alterations of the DI or the host’s surrounding tissues. We detected no surface alterations on DI, exception made one case, in which decreased Ti values (up to 60%) were found. However, in this case the decrease in Ti was associated with the technique used for implantoplasty (4). Histopathological study showed no Ti particles on the tissue surrounding the implants. As no alteration in Ti values was detected in other cases it is possible to discard a clear relation between the oncological lesion and deterioration of the implant surface.

The relation between peri-implantitis and oncological lesions continues to be feeble (6). Although there is insufficient epidemiologic evidence to establish a link to a specific oncogenic risk factor, a previous study suggested that chronic inflammation or peri-implantitis could increase the likelihood of oncological mutations in patients with premalignant lesions such as leukoplakia, erythroplakia, oral lichen planus, stomatitis or tobacco-related keratosis (8-10). Other oncological factors such as tobacco and alcohol, were detected in two of our newly-detected oncological cases, but these same factors have been linked with the development of peri-implantitis. The time of appearance or recurrence of a malignant lesion after DI placement appears to vary from 5 months to 8 years (11,13). Regrettably, this large time lapse impedes establishing a cause-effect relation between the two variables. Some authors like Albrektsson *et al.* (14) have stated that carcinoma lesions have been previously mistaken as peri-implantitis.

Indeed, peri-implantitis, by definition, involves not only mucosal damage but also damage to the bone surrounding the implant as well (12). The association of peri-implantitis with other patient-related risk factors, such as gingivitis and smoking, could act as a triggering factor of an oncological process (11). 

Our findings are in consonance with those presented by Singhvi *et al.*, including DI cases associated with oral cancer, though no direct etiological relation with DI was evident. Nevertheless, in their review, Singhvi *et al.*, noted chronic trauma as a paramount factor in association with ill-fitting dentures. An inflammatory response associated with trauma represents a common factor with the peri-implantitis observed in our series. Likewise, Moergel *et al.*, cited by Javed *et al.* in 2012, presented the largest series of cases with cancer near DI, emphasizing the role of peri-implantitis as a predominant clinical sign in 11 of 26 cases, including one from their own series and ten from other authors (9). Peri-implantitis as a chronic inflammatory process provides an environment with cytokines as inflammatory mediators with antitumor and protumor effects. This chronic inflammation may be a risk factor for cancer, though no direct relation has yet been shown.

The setting of maximum risk could be a previously radiated patient with a genetic profile favoring CA lesions who undergoes dental rehabilitation with implants over bone lacking regenerative ability and where peri-implantitis is likely (15).

After a complete review of the literature and the study of our clinical cases we conclude that, there is still no definitive evidence for DI as a direct risk factor for SCC. However, chronic irritators of the oral mucosa have been postulated as risk factors due to their presence in cases of oral cancer, and DI are frequently related with peri-implantitis, a chronic trauma of oral mucosa that is increasing. Given the presence of trauma and inflammatory processes close to DI; these could be contributing factors in oncological disease. Histological analysis confirmed the presence of peri-implantitis in all the cases studied. An extensive series of cases with a longer follow-up is advised to establish a possible direct etiologic or contributory agent for oral cancer.

## References

[1] Kim L, King T, Agulnik M (2010). Head and neck cancer: changing epidemiology and public health implications. Oncology (Williston Park, NY).

[2] Singhvi HR, Malik A, Chaturvedi P (2017). The Role of Chronic Mucosal Trauma in Oral Cancer: A Review of Literature. Indian Journal of Medical and Paediatric Oncology: Official Journal of Indian Society of Medical & Paediatric Oncology.

[3] Mendes FA, Borges T de F, Gonçalves LC, de Oliveira TR, do Prado CJ, das Neves FD (2016). Effects of new implant-retained overdentures on masticatory function, satisfaction and quality of life. Acta Odontol Latinoam.

[4] Martins O, Polares Baptista I (2015). Implantoplasty approach on peri-implantitis - case series. Europerio.

[5] Moy PK, Medina D, Shetty V, Aghaloo TL (2005). Dental implant failure rates and associated risk factors. Int J Oral Maxillofac Implants.

[6] Salgado-Peralvo AO, Arriba-Fuente L, Mateos-Moreno MV, Salgado-García A (2016). Is there an association between dental implants and squamous cell carcinoma?. Br Dent J.

[7] Raiser V, Abu-El Naaj I, Shlomi B, Fliss DM, Kaplan I (2016). Primary Oral Malignancy Imitating Peri-Implantitis. J Oral Maxillofac Surg.

[8] Bhatavadekar NB (2012). Squamous cell carcinoma in association with dental implants: an assessment of previously hypothesized carcinogenic mechanisms and a case report. J Oral Implantol.

[9] Javed F, Al-Askar M, Qayyum F, Wang HL, Al-Hezaimi K (2012). Oral squamous cell carcinoma arising around osseointegrated dental implants. Implant Dent.

[10] Bhandari S, Rattan V, Panda N, Vaiphei K, Mittal BR (2016). Oral cancer or periimplantitis: A clinical dilemma. J Prosthet Dent.

[11] Block MS, Scheufler E (2001). Squamous cell carcinoma appearing as peri-implant bone loss: a case report. J Oral Maxillofac Surg.

[12] Marini E, Spink MJ, Messina AM (2013). Peri-implant primary squamous cell carcinoma: a case report with 5 years' follow-up. J Oral Maxillofac Surg.

[13] Schache A, Thavaraj S, Kalavrezos N (2008). Osseointegrated implants: a potential route of entry for squamous cell carcinoma of the mandible. Br J Oral Maxillofac Surg.

[14] Albrektsso T, Dahlin C, Jemt T (2014). Is marginal bone loss around oral implants the result of a provoked foreign body reaction?. Clin Implant Dent Relat Res.

[15] Gallego L, Junquera L, Baladrón J, Villarreal P (2008). Oral squamous cell carcinoma associated with symphyseal dental implants: an unusual case report. J Am Dent Assoc.

